# Suspension of Care for Patients With Spasticity During COVID-19 Pandemic: Ethical and Medico-Legal Point of View Starting From an Italian Study

**DOI:** 10.3389/fmed.2021.754456

**Published:** 2021-11-30

**Authors:** Antonio De Donno, Adriano Acella, Carmelinda Angrisani, Giulia Gubinelli, Gianluca Musci, Gianluca Gravili, Chiara Ciritella, Andrea Santamato

**Affiliations:** ^1^University of Bari-Section of Legal Medicine, Policlinico di Bari Hospital, Bari, Italy; ^2^University of Foggia-Spasticity and Movement Disorders Unit, Physical Medicine and Rehabilitation Section, Policlinico Riuniti, Foggia, Italy

**Keywords:** COVID-19, chronically ill, spasticity, patient safety, medico-legal issues, telemedicine

## Abstract

The COVID-19 pandemic has revolutionized the habits of entire communities, having even more profound negative effects on assistance for the chronically ill. The sudden demand for extraordinary resources caught all worldwide countries unprepared, highlighting shortages in provision of care services. This applies to all patients, affected by COVID-19 or not, as many need continuing access to chronic diseases treatments. Almost all of the energy available has been directed toward care of COVID-19 patients, and almost nothing has been done to continue therapy for patients with spasticity. This study builds on a recent article and discusses its results as a basis for highlighting the ethical dilemmas and unintended consequences of health systems changing their priorities during the pandemic. The above mentioned study has shown increased patient-perceived spasticity during lockdown (72.2%) with reductions in perceived quality of life (70.9%). Telemedicine tools have proved insufficient, with access by only 7.3% of these patients. Despite the health emergency, it cannot be denied that this situation is a violation of these patients' rights and dignity. The healthcare system will also have to bear increased costs in the future to recover the loss of previous therapies benefits, because of their interruption. The real challenge will be to exploit the critical issues emerged during the pandemic, and to resolve the measures needed to take the care to the patient, and not *vice versa*. This applies particularly to fragile patients, to respect their dignity and right to care.

## Introduction

The influence of the COVID-19 pandemic goes well-beyond the evident immediate clinical impact on the entire community. The Italian Government has made substantial economic and organizational efforts to guarantee staff resources, hospital beds, and ICU facilities for patients with ARDS caused by SARS-CoV-2 related pneumonia (1, 2). SIAARTI and SIMLA have issued guidelines, with respect to the ethical dilemmas lived daily by the practitioners, which acknowledge that under exceptional circumstances, the choice to treat only patients with greater chances of therapeutic success may ultimately be justified (3). COVID-19 pandemic had profound effects on both daily habits and behaviors of all populations, as well as on the assistance provided to the chronically ill (4). Most health resources have been, and still are, directed into managing the “COVID-19 emergency.” This “monopolization” of resources has understandably had repercussions for the management of patients with chronic diseases (4).

Indeed, the management of outpatient and inpatient hospital activities has changed globally (5), due to continuous updating of national regulations. Although on the one hand these measures have been in response to the urgent need to reduce overcrowding in hospitals and to contain the contagion of patients and healthcare personnel (6), on the other hand, in our opinion, they have raised many critical issues (7), mainly in terms of equity of distribution of healthcare treatments. Should an efficient health system postpone an effective health service to maintain or improve the health of the many? Is it right to overshadow the quality of life of patients with chronic diseases or disabilities? Does this mean that we are willing to accept the deterioration in the health of these minorities?

In many European, and also non-European, contexts, the provision of telematic care [i.e., so-called “telemedicine” (8)] has been proposed to counter the suspension of outpatient services during the pandemic (8), to guarantee a continuum of the diagnostic–therapeutic management of patients. The definition adopted in 1997 by the World Health Organization (9), indicates that telemedicine is “the provision of care and assistance services, performed at a distance through the use of information and communication technologies to promote global health, controlling pathologies and health care, as well as education, training, and management of health personnel and research in the interests of the health of the individual and the community” (9). Together with China, which was the first country hit by the COVID-19 pandemic, the provision of telemedicine in many countries has recently undergone sudden and large acceleration, to complement the traditional care models.

According to Alex Jadad, the founder of the Center for Global Health Innovation at the University of Toronto, “Whether I am deep in Malawi or deep in the Amazon, all I need is a mobile phone and a connection that allows me to speak with a doctor. It is all that is required for a clinical evaluation” (10). However, is that really all that it takes for a clinical evaluation? Can it be considered sufficient for a good healthcare service? In part, probably yes, if we refer to simple follow-up situations. In the case of patients who need drugs defined exclusively for hospital use (which therefore cannot be administered at home), what impacts do the deferral of treatments and the provision of remote services have? Moreover, from the emotional point of view, what is the patient's perception of this level of safeguarding of their health, and of the quality of the doctor–patient relationship?

A recently published Italian study that relate to the suspension of gold-standard therapies focused on botulinum toxin (BoNT-A) injections for spasticity treatment. That provided some food for thought (11).

## Case Description

In compliance with the Italian national regulation published on 16 March 2020, outpatient and “non-urgent” hospitalization activities were reshaped. This had a dual purpose: to make significantly more beds available for COVID-19 patients, and to limit the infection among patients (12).

Clinical evidence of the role of BoNT-A in the treatment of spasticity is well-supported (13–17). This neurotoxin helps to prevent deformities, improves motor function, and relieves symptoms in subjects suffering from spasticity of various etiologies. It acts through reduction of muscle hyperactivity, pain, contractions, with consequent alterations to patient mobility (e.g., balance, walking, and use of limbs) and personal care (e.g., hygiene, eating, and dressing), and helps in the use of aids (e.g., orthoses, wheelchairs). Removal of BoNT-A use and availability thus has clear repercussions on the quality of life of these patients (18).

The study taken into consideration was conducted when the need to contain the spread of the virus and to maintain social distancing in Italy led to the temporary suspension of BoNT-A treatment in the outpatient setting (11).

The patients were recruited consecutively from outpatient departments of four Italian spasticity centers. A phone-based survey was administered to 151 patients who suffered from various kinds of spasticity derived from traumatic brain injury or ischemic/hemorrhagic stroke and had been treated for at least 1 year with periodic injections of BoNT-A (Table 1).

**Table 1:** Characteristics of participants (11).

	***N* (%)**	**Years (mean ± SD)**
**Participants (N)**	151	
**Gender**		
Male	90 (59.6)	
Female	61 (40.4)	
**Age group (years)**		58.42 ± 14.64
18–40	18 (11.9)	
41–60	59 (39.1)	
61–80	74 (49)	
**Time since event**		7.81 ± 7.34
**Disease**		
Ischemic stroke	75 (49.7)	
Haemorragic stroke	48 (31.8)	
Traumatic brain injury	28 (18.5)	
**Paretic side**		
Left	80 (39.7)	
Right	60 (53)	
Both	11 (7.3)	
**Time since first injection (years)**		3.07 ± 1.03
1–2 years	51 (33.7)	
2–3 years	27 (17.9)	
More than 3 years	73 (48.3)	
**Affected limb**		
Upper limb	21 (13.9)	
Lower limb	16 (10.6)	
Both	114 (75.5)	

*Data are reported as mean ± SD. SD, standard deviation; N, number*.

The aims were to evaluate the effects of the discontinuation of these BoNT-A treatments and the implications of this disruption on quality of life, as perceived by patients (11).

For the patients involved, most perceived increases in their spasticity (72.2%), and in related symptoms, such as pain, and in their quality of life (Table 2). Further agreement was seen for their perception of the importance of BoNT-A treatments, which were considered essential by 99.3% of the sample. The large majority of patients considered the suspension of treatment inappropriate (77.4%) (11).

**Table 2:** Covid-19 related factors, rehabilitation and quality of life impact on spasticity and independence (11).

**Item**	**“Worsening of spasticity”**				**“Worsening of independence”**			
	**No**	**Yes**			**No**	**Yes**		
	***N* (%)**	***N* (%)**	***p*-value (*r*)**	**Sig**.	***N* (%)**	***N* (%)**		**Sig**.
**Mood**								
At all	8 (30.8)	18 (69.2)	<0.001 (0.138)	HS	15 (57.7)	11 (42.3)	<0.001 (0.304)	HS
Mild/discrete	28 (32.9)	57 (67.1)			49 (57.6)	36 (42.4)		
Significant/extreme	6 (15.0)	34 (85.0)			7 (17.5)	33 (82.5)		
**Sleep**								
At all	22 (36.7)	38 (63.3)	0.074 (0.183)	NS	34 (56.7)	26 (43.3)	0.003 (0.228)	HS
Mild/discrete	18 (24.7)	55 (75.3)			35 (47.9)	38 (52.1)		
Significant/extreme	2 (11.1)	16 (88.9)			2 (11.1)	16 (88.9)		
**Relationships**								
At all	14 (38.9)	22 (61.1)	0.213 (0.094)	NS	18 (50.0)	18 (50.0)	0.022 (0.153)	S
Mild/discrete	20 (23.3)	66 (76.7)			46 (53.5)	40 (46.5)		
Significant/extreme	8 (27.6)	21 (72.4)			7 (24.1)	22 (75.9)		
**Community life**								
At all	11 (39.3)	17 (60.7)	0.087 (0.179)	NS	13 (46.4)	15 (53.6)	0.007 (0.169)	HS
Mild/discrete	24 (30.0)	56 (70.0)			46 (57.5)	34 (42.5)		
Significant/extreme	7 (16.3)	36 (83.7)			12 (27.9)	31 (72.1)		
**Motivation**								
At all	11 (31.4)	24 (68.6)	0.222 (0.144)	NS	18 (51.4)	17 (48.6)	<0.001 (0.229)	HS
Mild/discrete	26 (31.0)	58 (69.0)			48 (57.1)	36 (42.9)		
Significant/extreme	5 (15.6)	27 (84.4)			5 (15.6)	27 (84.4)		
**BoNT-A QoL**								
No	22 (50.0)	22 (50.0)	<0.001 (0.317)	HS	37 (84.1)	7 (15.9)	<0.001 (0.476)	HS
Yes	20 (18.7)	87 (81.3)			34 (31.8)	73 (68.2)		
**Rehabilitation**								
No	22 (24.4)	68 (75.6)	0.331 (−0.106)	NS	33 (36.7)	57 (63.3)	0.003 (0.277)	HS
Physical activity or mobilization	15 (30.0)	35 (70.0)			29 (58.0)	21 (42.0)		
Home or telerehabilitation	5 (45.5)	6 (54.5)			9 (81.8)	2 (18.2)		

*Data are reported as number (%); N, number of answers; HS, highly significant; S, significant; NS, not significant; Sig., significance; r, Spearman rho correlation; BoNT-A QoL, botulinum neurotoxin type A discontinuation quality of life*.

The interviewed subjects also reported significant reduction in the skills previously acquired for walking, and the use of the arm and hand. Personal care management also worsened, due to increased difficulties with washing, dressing and eating. These deficits have clear repercussions on their quality of life and functional independence, and they were significantly more relevant in severe spasticity patients. Consequently, they complained of greater difficulties in their daily use of aids for walking or wheelchair use, and 53% also reported worsening of autonomy (Table 2) that led to greater need for caregiver assistance (11).

The data on the continuation of motor rehabilitation in the lockdown phase are particularly fascinating and discouraging. In the Italian study, only 7.3% of the patients carried out telerehabilitation or home rehabilitation with professionals. The reasons for this failure might be related to a lack of availability of these IT tools, or their ability to use them, or to the distrust in these tools held by professionals or these patients. Social and economic differences in the population also have severe negative repercussions in the equal distribution of care.

## Discussion: Defining Issues and Ethical Analysis

One of the fundamental values and the universally recognized ethical principle of *Ars Medica* is the protection of life and physical and mental health of man, and the relief from suffering, while respecting the freedom and dignity of the human person (19). However, the study examined show that a significant proportion of patients experienced increases in pain symptoms following the discontinuation of their BoNT-A treatment. The suffering of these patients due to the increased pain thus poses a difficult ethical and medico-legal questions that cannot be further ignored. Even more so, if we consider the consequent reduction in functional independence of these patients, and increase in their need for daily and ongoing assistance. Therefore, the purposes of the medical acts are only met in terms of containing the infection, while falling far short in terms of respect for a person's inviolable rights and the patients' dignity.

In addition to the problems analyzed here, it is also necessary to consider the possibility that the suspension of rehabilitation and drug therapies is also inducing a state of “regression” of the clinical picture, with a loss of the therapeutic benefits obtained previously, thus jeopardizing greater subsequent patient benefits. To prevent this unacceptable clinical scenario, a further socio-sanitary, economic, and human effort is necessary and urgent.

In recent decades, the concept of individual care has been increasingly focused on the quality of life of the patient, and so enhancing a series of aspects that go beyond the idea of health in a clinical sense. This includes “the individual's perception of one's position in life in the context of the cultural systems and reference values in which it is inserted, and about one's goals, expectations, standards, and interests” (20). Therefore, when we talk about patient quality of life, we take into consideration issues that go beyond the individual's “physical health” conditions. Instead, we must include cultural, subjective, and inherent aspects of the physical, social, and psychological well-being of the patient, with the related effects on patient emotions and awareness that any specific pathology has.

Spasticity has a significant and inevitable negative impact on various daily aspects of the dynamic–relational and psychological profile of the patient. However, provided they are constant, motor rehabilitation associated with pharmacological therapy can significantly benefit the patient, allowing the limits imposed by their neurological pathology to be at least partially overcome. If the burden of psychic and physical effects resulting from the therapeutic suspension is attached to this delicate substrate, it is easy to understand the extent of treatment removal on the patient quality of life. The study mentioned here has shown clear negative psychological implications related to BoNT-A administration, where the majority of these patients find this therapy helpful, thus arguing that it should not be interrupted or postponed. The caregiver is also of crucial importance in managing the daily life of a patient with spasticity, as this figure compensates for the limits imposed by the reduced functional independence, with an essential role in the psyche of the patient.

The reduction in autonomy related to the adverse effects of withdrawal of these BoNT-A treatments has led to an increased need for assistance from caregivers, thus creating further disparity of opportunities based on financial resources of the patient. If the caregiver is not a family member who lives with the patient, it will be necessary to hire qualified personnel to assist, with the consequent increase in the family expenses. Also, functional deficits are associated with reduced work skills and the resultant reductions in any earnings related to the professional activity of the patient.

It is fair to ask what might be the wisest choice: whether to protect life at the expense of repercussions on psychophysical well-being, or whether to ensure continuation of outpatient services while accepting the increased risk of contagion, which therefore puts life itself at risk. In the context of protection of the community, the closure of these outpatient services represents a safeguarding strategy, to limit the spread of the SARS-CoV-2 virus; however, as mentioned, this has considerable impact on the quality of life of fragile patients affected by chronic diseases.

Therefore, we must question the need to guarantee an inviolable right (i.e., the health of the citizen) while safeguarding the well-being of the community. A partial response comes from the implementation of telematics systems, which have provided, if only in part, a continuum in the diagnostic–therapeutic management in the new pandemic scenario.

## Telemedicine: Limits and Future Perspectives

Since the beginning of the 21st century, technological and media development has led to an increase in interest in “remote medicine,” as the so-called telemedicine, to improve medical care access, especially in rural areas (21, 22). Then, the emergency of the COVID-19 pandemic ushered in a new era for telemedicine (23, 24), with the consequent changes to the provision of the healthcare services. Here, outpatient visits were rapidly converted, where possible, into virtual visits (25).

In times of health emergency, two priorities have been identified. The first is to ensure home care for people affected by COVID-19 and for those who, although not infected, present the need for home care due to their pathological conditions or frailty. The second priority is to protect healthcare personnel as much as possible from the risk of contagion (26). In Italy, the *Istituto Superiore di Sanità* issued their COVID-19 Report N° 12/2020 (Interim indications for telemedicine assistance services during the COVID-19 health emergency), to thus promote the use of health services electronically, as well as for out-of-hospital management of COVID-19–positive cases, and also for management of chronic diseases.

Indeed, an undeniable advantage of telemedicine is the possibility to quickly reach the patient through the now commonly used electronic tools (e.g., smartphones, PCs, tablets). This facilitates the possibility of multi-specialist assessments (27), as well as making clinical comparisons between different specialists more immediate, and including the general practitioner. Albeit potential and independent of the pandemic context, another advantage is that waiting lists can be streamlined for those conditions that do not require a face-to-face visit with a doctor or nurse, with savings in terms of social and economic costs (28). Public health authorities introduced mobile apps which can be used in clinical practice to monitoring the patient's chronic condition (29).

Some studies have highlighted the social discrimination induced by telemedicine, as it excludes the lowest classes in society. Furthermore, it can often be of limited effectiveness because of the relatively limited functionality of the current telematics systems in supporting a remote medical path (30). On the contrary, however, the users can benefit from reduced transfer times to and from clinics, and also the reduced associated costs, with the possibility of these resources being reinvested in home therapies (31). We also note that remote assistance reconsider the use of resources in health centers (15).

A possible concern here is the concept of remote physical rehabilitation treatments. The Italian study (11) showed that the number of patients with spasticity who joined telerehabilitation services was very low. Indeed, during the lockdown at the time, 92.7% of the patients were left without any rehabilitation treatment at all, an important statistic whether or not this was due to unavailability of means or skepticism of specialists or patients toward telematic medicine.

Therefore, it will be necessary to promote telemedicine and resolve the unsolved ethical and medico-legal problems for which there remain no specific legislation. In 2012 in Italy, National Guidelines were introduced by the Ministry of Health on the processing of personal data and the acquisition of informed consent, as specifically the need to give consent only at the beginning of a treatment, or possibly to renew it for every single telematic service in the follow-up area.

From an ethical point of view, there is the risk of worsening doctor–patient relationships, with the consequent reduction in patient compliance due to a depersonalization of the medical acts. This has inevitable repercussions on the quality of these relationships and on the therapeutic “alliance” between doctor and patient, as well as in terms of the outcome of any treatment. Indeed, a more immediate and tangible consequence of the lack of physical interaction between doctor and patient is the loss of direct objective evaluation of the patient. This represents the real and insurmountable limit of telemedicine, as although technology allows a shortening of the distance between doctor and patient, it inevitably does not allow direct physical contact. Then there is the impossibility of direct administration of drugs that are intended exclusively for hospital use or are only administered by a doctor or nurse; indeed, such as BoNT-A for the patient with spasticity.

Therefore, face-to-face access to those categories of patients who will benefit from physical diagnosis or who require periodic drug administration should still be guaranteed. On the other hand, remote physiotherapy can be implemented through telemedicine, as this can be performed independently or with the help of a caregiver, again with the advantages of reduced travel times and specialized transport costs.

In these terms, telemedicine does not represent a solution that can deal with all of the problems (32). It appears, instead, not to be an exhaustive option, as there are some medical acts in which the presence of a healthcare professional is essential (33). It does suggest, however, the need to modify and implement local social assistance resources, to make access to drug treatments more possible, even in the post-pandemic period.

On the other hand, the use of telematic tools for the follow-up of chronic diseases, such as diabetes and metabolic disorders, appears to be more promising. Here, the diagnosis has already been carried out, and telemedicine allows the progress of patients to be followed without any particular limitations, avoiding the need for the presence of a doctor or nurse. Also, the use of “apps” can be aimed at better self-management of chronic patients (1).

## Conclusion of the Case and Comment

The current pandemic has required significant changes in the organization of healthcare and its facilities, and the management of both inpatient and outpatient hospital activities. It has highlighted significant ethical and medico-legal problems relating mainly to the suspension of treatments for spasticity, or other disability or chronic disease, which has shown clear deterioration in the quality of life perceived by the patient. At the same time, the COVID-19 “tsunami” has forced organizational innovations in healthcare processes, through rapid implementation of remote telemedicine.

While highlighting the incredible social and economic advantages that make telemedicine a useful tool in a post-pandemic perspective, this cannot alone provide an exhaustive option for the management and care of patients suffering from chronic pathologies, such as spastic patients, who need BoNT-A direct administration. The evaluations examined here allow us to affirm that flexible integration of this tool with traditional management practices is necessary. Finally, the occasion appears propitious for the restyling of territorial medicine (i.e., out of hospital), with a decisive intervention that favors the movement of socio-health treatment of primary chronic diseases, to decongest hospital activities in line with the need to contain and rationalize healthcare costs.

The real challenge will be to exploit the critical issues that have emerged in this pandemic period, and to define the best remedial measures to take the care to the patient, and not *vice versa*. During this, particular attention must be paid to fragile patients, with full respect for their dignity, along with promotion of the right to care for all those who are sick.

## Data Availability Statement

The original contributions presented in the study are included in the article/supplementary material, further inquiries can be directed to the corresponding authors.

## Author Contributions

AD and AS had the idea of writing the manuscript. AA, CA, GGu, GM, GGr, and CC co-drafted and wrote the manuscript. All authors have read and agreed to the published version of the manuscript.

## Funding

The publication of this article has been financed by University of Foggia.

## Conflict of Interest

The authors declare that the research was conducted in the absence of any commercial or financial relationships that could be construed as a potential conflict of interest.

## Publisher's Note

All claims expressed in this article are solely those of the authors and do not necessarily represent those of their affiliated organizations, or those of the publisher, the editors and the reviewers. Any product that may be evaluated in this article, or claim that may be made by its manufacturer, is not guaranteed or endorsed by the publisher.

## Abbreviations

ICU: intensive care unit
ARDS: acute respiratory distress syndrome
SIAARTI: Italian Society of Anesthetics and Reanimation
SIMLA: Italian Society of Legal Medicine
BoNT-A: botulinum toxin.
